# From across the Seas

**Published:** 1917-04-28

**Authors:** 


### FROM ACROSS THE SEAS.

### Hospital Costs.

An interesting matter has been started by Mr. J. E. Mackey, M.L.A., who, accompanied by the Inspector of Charities (Mr. Malcolm), on February 2 visited Warragul, a town at the junction of the main Gippsland Railway with a branch line, about three hours' run from Melbourne, for the purpose of "opening" the new operating theatre just added to the Warragul District Hospital at a cost of about £1,000. Mr. Mackey improved the occasion by remarking upon the relative difference of the annual cost per bed and per patient of Warragul and Melbourne—£65 and £4 1s. against £136 and £7 3s. for the year ending June 30, 1916. Melbourne's average of occupied beds is 357, with 31,643 out-patients and casualties, and a large clinical school, while Warragul Hospital has an average of twenty-four occupied beds, no out-patients, and no clinical school. The Gippsland Hospital (Sale), beginning the same term with a balance of £120 12s., had a gross revenue of £4,173 12s. 10d., finishing the year with a balance of £573 9s. 11d., for which 707 in-patients and 143 out-patients were treated; cost per bed and per patient being set down at £63 5s. and £5 1s. 7d. respectively, the highest, it may be remarked, in its fifty years' existence.

Mr. Mackey's remarks were immediately answered by Sir John Grice, president of committees at Melbourne Hospital, who quoted from Sir Henry Burdett's book, "The Uniform System of Accounts, Audit, and Tenders for Hospitals and Institutions," the passage commencing "It must further be borne in mind that the bare statement that a bed costs so many pounds at one institution, and half as many pounds at another, does not necessarily, if at all, determine that the management of the one is infinitely superior to the other, or *vice versa*," etc. In replying, Mr. Mackey sticks to his thesis that the larger the number of patients, the less should be the cost. "Big business should mean cheap business." And he points out that the cost of provisions only at Melbourne is £25 4s. per bed, and at Warragul £19 10s., although Melbourne has the advantage of large purchases made at the lowest rates, while Warragul has the disadvantage of small purchases, with railway freight to pay on the bulk of its supplies. In this connection Gippsland Hospital shows better than either, as the cost of provisions seems to work out at about £18 13s. per bed. Country hospitals have this great advantage over town in the three items of bread, meat, and milk being practically at their doors.

### Finance of a Joint Stock Hospital.

The Western Minnesota Hospital at Graceville represents an investment of £3,800, the greater part of which was raised by money advanced by stockholders, and the remainder—£800—was given by charitable persons. The first annual report was recently issued, and the finance of the institution is described in an article by Dr. C. I. Oliver in *The Modern Hospital* for February. The highest bed capacity is twenty-one, and the average occupation seventeen; within a year 426 patients were admitted and paid fees amounting to £1,945, outstanding accounts bringing this sum up to £2,048. The outgoings, £1,693, included a fairly substantial sum for permanent improvements. It is not stated whether the building is a new one, but from the smallness of the capital and from the appearance of the institution as depicted in a photograph, it may be judged that this is not so. As a result of the year's working the stockholders have been paid a dividend of 10 per cent. There are six single rooms in which the patients pay £5 per week, in five others the fee is £4 per week, and three two-bed wards bring in £3 a bed. The operation fee is £1, and the fees thus raised are the entire source of income. The patients arrange medical fees with the doctors, who supply their own anæsthetists and pay them. The doctors have to guarantee the hospital bill for all patients they bring in. There is a small training school for nurses, and four pupil-nurses are taken who receive £1 each per month, with board, room, and uniforms; the superintendent nurse draws an annual salary of £192.

